# Lessons on multicellular two- (2D) and three-dimensional (3D) culture in parasitology: Insights, challenges and future directions

**DOI:** 10.1016/j.ijpara.2025.12.004

**Published:** 2025-12-16

**Authors:** David Smith, Matias G. Perez, Miriam E. Garner, William M. Anderson, Collette Britton, Maria A. Duque-Correa, Nichola E.D. Calvani

**Affiliations:** a Moredun Research Institute, Pentlands Science Park, Penicuik, United Kingdom; b School of Biodiversity, One Health and Veterinary Medicine, University of Glasgow, Glasgow, United Kingdom; c School of Biological Sciences, Queen’s University Belfast, Belfast, United Kingdom; d Cambridge Stem Cell Institute, University of Cambridge, Cambridge CB2 0AW, United Kingdom; e Sydney School of Veterinary Science, Faculty of Science, The University of Sydney, Australia

**Keywords:** Protozoa, Helminth, Host-parasite interactions, *In vitro* infection, Organoids, Spheroids

## Abstract

Advances in multicellular two-dimensional (2D) and three-dimensional (3D) cell culture systems are providing parasitologists with new tools to investigate host–parasite interactions *in vitro*. These models offer tissue-specific and, increasingly, host-specific alternatives to traditional 2D monoculture and animal systems, with applications across protozoan and helminth biology. Spheroids, organoids, and emerging assembloid platforms capture key aspects of tissue architecture and function, enabling co-culture of parasites and their products, including dynamic analysis of interactions at defined host interfaces. In recent years, these systems have been adapted to model infection processes, parasite development, immune modulation and tissue remodelling across a range of parasite taxa and tissue types, particularly of the gastrointestinal tract.

This review outlines applications of multicellular 2D and 3D cell culture systems in parasitology, drawing on examples from both human and veterinary research. We highlight lessons learned from published works to date that have accelerated the uptake and refinement of these approaches. We also examine technical challenges, including issues of standardisation, scalability, model accessibility, and species representation, particularly for livestock hosts.

Looking ahead, the integration of immune, stromal, and microbial components into these models, as well as advances in imaging and omics technologies, and CRISPR-Cas9-mediated engineering of host organoids, promise increasingly sophisticated platforms for studying parasite biology, host tissue responses and pathogenesis. With continued investment and cross-disciplinary collaboration, multicellular culture systems are poised to play a central role in reducing animal use, improving model predictiveness, and supporting the development of next-generation antiparasitic therapies and interventions, including drugs and vaccines.

## 1 Introduction

**Fig. 1:**
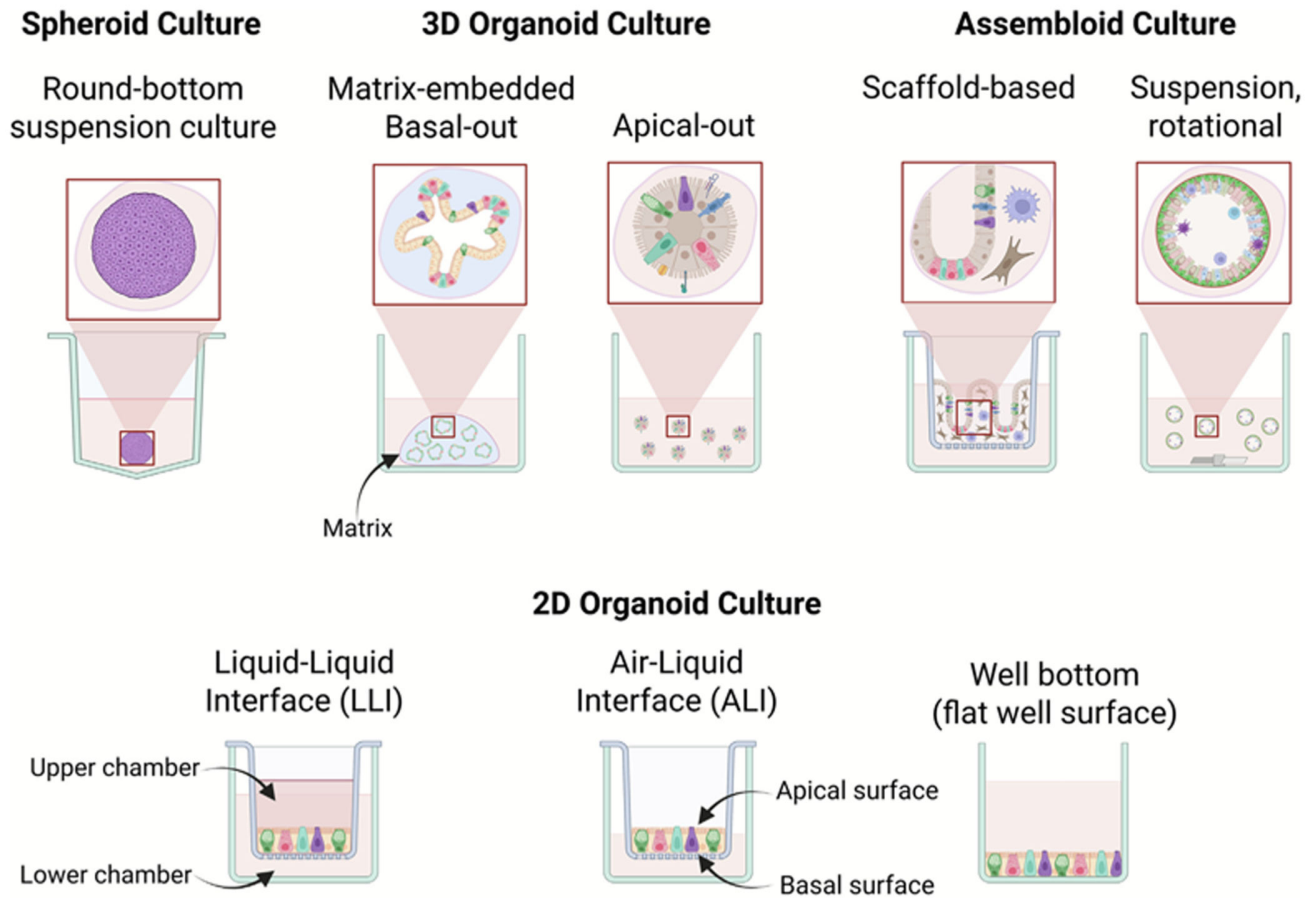
Overview of multicellular 2D and 3D cell culture systems used in parasitology research. Spheroids (left) are typically formed from immortalised or primary cells under low-attachment conditions. In contrast, organoid cultures (middle) are self-organising structures derived from stem or progenitor cells and can be embedded in extracellular matrix for basal-out orientation or cultured in suspension for apical-out configurations. Assembloid cultures (right) incorporate multiple cell types or tissues, allowing more complex architecture and functional integration. 2D organoid-derived monolayers (bottom) can be grown on transwell inserts in liquid–liquid interface (LLI) or air–liquid interface (ALI) formats to model epithelial barrier function and polarised interactions, or directly on well bottoms. These complementary approaches enable diverse experimental readouts, including apical vs basal stimulation, live imaging, and functional assays relevant to host-parasite interactions.

In recent years, advances in two- (2D) and three-dimensional (3D) cell culture techniques have enabled the development of increasingly sophisticated *in vitro* culture systems with relevance to the study of a variety of parasites. These systems have application to both protozoa and helminths of significance to human and animal health. The model systems span a continuum of complexity, ranging from **spheroids** (see Glossary), simple aggregates of one or more cell types that mimic elements of basic tissue architecture and function, to **organoids**, which are self-organising multi-cellular structures derived from epithelial stem cells that recapitulate key structural and functional features of specific organs (Corrò et al., 2020; Hofmann et al., 2022) (Fig. 1). While they do not currently fully recapitulate the tissue niche, both organoids and, to a more limited extent, spheroids retain aspects of cellular heterogeneity, structural integrity, and physiological function of the tissue of origin (Lancaster and Knoblich, 2014; Wei et al., 2024). More recently, **assembloids, organs-on-a-chip, and organismoids** have emerged as sophisticated platforms that integrate multiple organoid types or incorporate additional cell types (e.g. immune, stromal, or endothelial cells), offering a more complete approximation of tissue- or system-level complexity, albeit with increased cost and technical demands (Marx et al., 2021; Zhu et al., 2023; Papamichail et al., 2025) (Fig. 1). Together, these culture systems offer powerful new tools for investigating fundamental parasite biology, host-pathogen interactions and disease mechanisms, with potential to streamline drug/vaccine development by reducing our reliance on traditional animal models in alignment with the **three Rs** (the replacement, reduction and refinement of the use of animals in research). These efforts are reinforced by recent government initiatives such as Australia’s Commonwealth Scientific and Industrial Research Organisation (CSIRO) national roadmap for phasing in non-animal models, which identifies organoids and related *in vitro* technologies as critical tools for improving research reproducibility and reducing animal use across sectors (Arora et al., 2023). Similarly, the UK government recently announced a new strategy to deliver on a commitment to phase out animal testing in human research, underpinned by advances in 3D tissue culture technology, the Food and Drug Administration (FDA) have announced similar plans in the USA for monoclonal antibody and drug testing research, and the EU directive 2010/63/EU states the long-term goal of full replacement of animal testing “as soon as it is scientifically possible”.

The use of multicellular 2D and 3D cultures in parasitology builds on decades of foundational work in stem cell biology. A major turning point in 3D cell culture came in 2009, when murine **adult stem cells** (**ASCs**) were cultured to form intestinal crypt-villus-like structures *in vitro*, marking the beginning of organoid-based culture systems (Sato et al., 2009). This was then translated into the development of human intestinal organoids derived from patient biopsy samples (Sato et al., 2011). Since then, organoid technology has expanded rapidly across diverse tissue types and species, including canine, feline, equine, porcine, ruminant, bat, avian and reptilian species (Clevers, 2016; Hamilton et al., 2018; Post et al., 2020; Beaumont et al., 2021; Carey et al., 2021; Hellman, 2021; Nash et al., 2021; Smith et al., 2021; Calà et al., 2023; Chen et al., 2023; Zdyrski et al., 2024). The capacity of these systems to support extended co-culture of pathogens *in vitro*, while maintaining host-specific traits, makes them particularly valuable for studying parasites − including those with life cycles that are otherwise unsuited to *in vitro* maintenance.

This review highlights recent advances in multicellular 2D and 3D cell culture methods and their application to parasitology. It focuses on lessons learned from: (1) current applications in parasitology and other health and disease research fields, (2) present challenges and limitations in the uptake of these technologies; and (3) provides suggestions for future directions and priority areas specific to the study of parasitology. By leveraging these systems, researchers can gain deeper insights into the mechanisms of parasite invasion, growth and development, host-parasite interactions, tissue tropism, and host specificity, ultimately paving the way for the discovery of novel therapeutic strategies with applications to both human and veterinary medicine.

## 2 Lesson 1: Current applications of multicellular 2D and 3D cell culture systems in parasitology and other health and disease research fields

### 2.1 Spheroids, organoids and their relevance to parasitology research

#### 2.1.1 Spheroids

Spheroids offer a simple yet powerful intermediate between traditional 2D cell culture systems and more complex 3D organoids for modelling host-parasite interactions. As self-assembled cellular aggregates, spheroids better mimic *in vivo* tissue architecture and function than traditional 2D culture by enabling enhanced cell–cell and cell-matrix interactions, thereby preserving differen-tiated phenotypes and supporting studies on physiologically relevant responses to a range of parasites over extended periods (see Fig. 1) (Velasco et al., 2020; Ellero et al., 2021; Yang et al., 2023b). Their commercial availability, relative simplicity, scalability, and compatibility with standard multi-well formats make them particularly well suited to parasitology research, where cost, reproducibility, and experimental throughput are key considerations.

Spheroids are commonly generated from immortalised commercially available cell lines such as HepG2 and HepaRG (hepatocytes), Caco-2 (intestinal epithelium), or H69 (cholangiocytes), thereby supporting global standardisation and broader adoption across research groups (Velasco et al., 2020; Yang et al., 2023b). These models provide a reproducible foundation for both mechanistic and applied research and are especially valuable in more resource-limited settings or when high-throughput testing is required. Advanced options are now becoming commercially available, such as Gibco™ Human Spheroid-Qualified Hepatocytes, which consist of primary cells isolated from single donors. Unlike immortalised cell lines, these cells exhibit both phase I and II enzyme activity, albumin production, bile canaliculi formation, and can differentiate into both hepatocyte- and cholangiocyte-like lineages, providing a versatile platform for modelling hepato-biliary infections and local metabolic responses (ThermoFisher, 2025). While a range of human cell types for spheroid generation are commercially available, including liver, pancreatic islet (InSphero, 2025), skin (Merck, 2025), and cardiac models (NovoHeart, 2025), there are currently no non-human cell sources available for purchase. As advanced culture options from a range of species and tissue types are increasingly developed, physiologically-relevant 3D models should be actively biobanked (through research repositories or commercialisation), thereby supporting their broader uptake and use.

Multiple methods exist for generating spheroids, each with trade-offs in throughput, scalability, and structural control. Scaffold-free approaches such as **low-attachment plates, hanging drop cultures**, and **rotating wall vessel bioreactors** rely on the innate self-aggregation of cells in suspension (Fig. 1). The hanging drop method, for example, enables gravity-assisted aggregation and is widely used due to its simplicity and reproducibility across replicates (Yang et al., 2023b). Low-attachment plates exploit hydrophilic or neutral surfaces to prevent adherence, promoting uniform spheroid formation, often within a 5–7 day timeframe (Xing et al., 2024). Alternatively, **magnetic levitation** and **bioprinting techniques** have been applied to generate more complex or spatially organised spheroids, though these methods are typically more resource intensive. Scaffold-based methods incorporate **extracellular matrix (ECM)** mimetics or **hydrogels** to support cell adhesion and polarity, but can introduce variability in composition and may interfere with downstream analyses (Velasco et al., 2020) (Fig. 1).

These systems have been adapted across disciplines, including toxicology, cancer biology, pharmacology, regenerative medicine, and increasingly, infection biology. Spheroids represent a reproducible and cost-effective 3D model system with unique advantages for studying host-pathogen dynamics. Moreover, spheroids can be cultured with other cell types, such as endothelial cells, fibroblast and erythrocytes, to form multi-component co-culture systems (Dorst et al., 2014; Lazzari et al., 2018).

#### 2.1.2 Organoids

Organoids are typically derived from stem cells, which are undifferentiated cells with the unique ability to self-renew and differentiate into multiple cell lineages (Weissman, 2000; The Moredun Group, 2024). Organoids can be derived from several types of stem cells, including **embryonic stem cells (ESCs), induced pluripotent stem cells (iPSCs)**, and ASCs. Depending on the tissue, they can also be derived from a progenitor cell type, such as alveolar type II pneumocytes in the lung, hepatic progenitor cells in the liver, or luminal progenitor cells in the mammary gland. The formation of organoids largely depends on the self-renewing properties of stem and progenitor cells and their ability to differentiate into the diverse cell types associated with the tissue-of-interest. Based on their self-organising properties, these cells form 3D structures that more closely resemble the complexity of their native tissue counterparts compared to spheroids and traditional cell lines (Yang et al., 2023b).

The most common types of organoid platforms are **submerged culture** and **air–liquid interface (ALI)** culture models (Fig. 1). The submerged culture method is the most frequently used, where cell clusters or single cells are placed in a culture dish often (but not always) embedded in ECM gels (Driehuis et al., 2020). Other variations of submerged culture include **liquid–liquid interface (LLI)** in which liquid media is placed both above and below the culture (using a transwell setup; Fig. 1). Three-dimensional organoid-derived 2D monolayers plated directly on the base of culture wells also remain in use for specific applications. Organoids can also be cultured without an extracellular matrix, thriving in suspension-based systems, especially in stirred or circulating bioreactor cultures (Ye et al., 2024).

The key distinguishing feature of a submerged culture is that the cells within the system are fully surrounded by the culture medium (Fig. 1). Where utilised, the ECM provides structural support and signalling cues, but is porous, allowing culture medium to reach matrix-embedded cells. Common ECM gels include basement membrane extracts, such as Matrigel^®^ and Geltrex™, which offer a rich substrate for cell adhesion and differentiation. Recently, alternative protein-based “scaffolds” such as invasin and biopolymers such as xanthan gum have also been found to be effective substrates for organoid cultivation (Narazaki et al., 2025; Wijnakker et al., 2025), particularly for transwell or multi-well plate cultures. **Expansive culture medium** used in submerged culture typically contains components such as advanced Dulbecco’s Modified Eagle Medium (DMEM)/F12, penicillin/streptomycin, GlutaMAX, HEPES, B27, N2, various growth factors including epidermal growth factor (EGF), hepatocyte growth factor (HPF) or fibroblast growth factors (FGFs), and signaling molecules, such as Wnt3A, Noggin, and R-spondin-1, which regulate stem cell maintenance, cell proliferation and differentiation (Pleguezuelos-Manzano et al., 2025). However, the exact composition of the medium may vary depending on the tissue or species from which the organoid is derived (Shankaran et al., 2021). Moreover, the removal or reduction of certain growth factors and signalling molecules (e.g. Wnt) in a **differentiation media** formulation can be applied to induce cellular differentiation.

For gastrointestinal (GI) epithelial organoids, which have been the most widely used tissue model in parasitology research to date, cells differentiate to form a single layer of epithelium surrounding a central lumen, with distinct polarization, where the apical side of the cells faces the lumen (Fig. 1). Multiple studies have demonstrated that polarity can be inverted following the gentle removal of organoids from the ECM, producing organoids with an accessible apical surface (Co et al., 2019; Li et al., 2020; Smith et al., 2021; Blake et al., 2022) (Fig. 1). Furthermore, it was recently demonstrated that apical-exposed organoids retain their multicellularity and transcriptional profiles associated with basal-out organoids (Chapuis et al., 2025). These advancements are particularly relevant to the study of GI parasites and their use should be dictated by the innate behaviours and locations of the parasites and stages of interest.

Three-dimensional organoids can also act as a source of stem cells for other *in vitro* models, such as **multicellular 2D monolayers** or more sophisticated **tissue engineering (TE) models** (Yang et al., 2023b) (Fig. 1). Organoid-derived monolayers (multicellular 2D cultures) can be established by enzymatically dissociating 3D organoids into fragments or single cells and then seeding them onto flat surfaces or transwells. This approach represents another way of accessing the apical (luminal) side of the epithelium, which is especially useful for investigating host-parasite interactions that naturally occur at this interface.

The ALI culture system is a variation of the LLI model, whereby cells are seeded onto a transwell with a porous membrane. Initially, medium is added to the upper chamber and to the underlying well, until a 2D layer of cells has become established. Then, media is removed from the upper chamber (forming the “air” interface) in an outer culture dish, allowing nutrients to only diffuse into the organoids from the porous base (the “liquid” interface) (He et al., 2024) (Fig. 1). This exposure to air at the apical surface is particularly relevant to certain organoid tissue types, such as lung, and can promote cell differentiation and viability. The ALI culture system also helps to create more organ-specific environments by mimicking aspects of the naturally varied oxygen gradients that exist *in vivo*.

### 2.2 Organoids and 3D cultures as models of protozoan infection

Protozoan parasites infect a range of host species and tissue types, many of which also have multiple and diverse predilection sites within the same host (Table 1). Prior to the development of 3D cell culture technology, and organoid culture in particular, *in vitro* models of protozoan infection were limited to simple 2D cultures using standard host immortalised cell lines, such as fibroblast, epithelial or endothelial monocultures (Ramírez-Flores and Knoll, 2021). Therefore, experimental models were restricted in their cellular and physiological relevance. However, since the emergence of organoid technology, a broad range of protozoan parasites of medical and veterinary importance have been studied in 3D cell cultures derived from diverse tissue types (Table 1). This includes apicomplexan and kinetoplastid parasites, ranging from *Plasmodium* spp. in liver spheroids, *Toxoplasma gondii, Cryptosporidium* spp. and *Giardia* spp. in intestinal organoids (Martorelli Di Genova et al., 2019; Wilke et al., 2019; Holthaus et al., 2020; Holthaus et al., 2022; Caygill et al., 2025), to *T. gondii* and *Trypanosoma brucei gambiense* in brain organoids, to name a few. A relatively recent review from Ramírez-Flores and Knoll (2021) provides a comprehensive overview of the transition from 2D to 3D cell culture models in apicomplexan research in particular. Therefore, this section of the current review will focus on highlighting the various ways spheroids and organoids have been applied in protozoan research to inspire future research directions, as well as discuss some more recent developments.

The types of studies that 3D cell culture systems have been used for in protozoan research can be categorised into the following:

1. Infection models and parasite life stage development *in vitro*
2. Models for host-parasite interactions
3. Protozoa as delivery systems

#### 2.2.1 Infection models and protozoan parasite life stage development in organoids

When Heo et al. (2018) demonstrated that human intestinal organoids could be infected with *C. parvum*, they also achieved a major breakthrough in *Cryptosporidium* research. For the first time, the entire enteric life cycle of this parasite, including asexual stages, sexual stage micro and macrogamonts, and infective fer-tilised oocysts could be recapitulated in a single *in vitro* system. Importantly, the oocysts produced in the organoid model were shown to be infectious in mice. Moreover, the authors showed that intestinal organoids cultured in differentiation media were more readily infected than organoids cultured in an expansive growth media. This finding suggests that the parasite may have a preference for particular epithelial cell types that can be recapitulated in intestinal organoids, although it is possible that a component of growth media that is not present in differentiation media could have suppressed the performance of the parasites. Nonetheless, their findings clearly demonstrate that the plasticity of organoids permits cultivation and enrichment of particular cell types, thereby providing a useful tool for promoting parasite development in the lab. Shortly following the achievements of Heo et al. (2018), Wilke et al. (2019) demonstrated that similar results could be generated in an open-format ALI 2D model derived from murine **enteroids**, thereby negating the need to microinject parasites into the lumen (the method used by Heo et al. (2018) to infect 3D organoids) to achieve an *in vitro* model of *C. parvum* infection and life cycle development. Moreover, by capitalising on the fact that male and female parasites could be produced in the *in vitro* ALI model, they demonstrated this model as an effective platform for performing genetic crossover and gene fitness experiments using CRISPR-Cas9 gene-edited parasites.

At a similar time, Martorelli Di Genova et al. (2019) reported that feline intestinal organoids could be used to develop *T. gondii* through enteric sexual life stages that are naturally specific to the feline small intestine (SI). However, while this study identified that low delta-6-desaturase activity and high linoleic acid levels (specific to the feline intestine) act as exogenous drivers of sexual stage development in this parasite species, it did not result in the production of viable infectious oocysts. This contrasts with what was shown to be possible for *C. parvum* in the previously mentioned intestinal organoid model. Since the authors showed that delta-6-desaturase inhibition and linoleic acid supplementation were not sufficient to induce viable oocyst production in organoids, but were sufficient to do so *in vivo* in mice (Martorelli Di Genova et al., 2019), the other environmental cues required to complete the development of *T. gondii* parasites into viable oocysts must be present in the murine intestinal tract. It seems that key stimuli are currently missing in standard organoid cultures to trigger the formation of viable oocysts. These differences provide valuable insights into the complex cues required for parasite development and warrant further investigation. Comparative investigations between organoids and *ex-vivo* tissue could shed light on the missing component(s), and whether supplementation into an organoid model would better mimic the cellular environment in which they naturally exist.

Whereas mammalian intestinal organoids have been established as a useful platform to support the complete life cycle of *C. parvum*, organoids derived from chicken SI primary tissue have now been used to establish the *in vitro* culture of *Eimeria tenella* through infective life stages (Teng et al., 2025). *E. tenella* sporozoites were shown to invade the organoids and develop through schizogeny and gametogony life cycle stages, with released oocysts identifiable in the cultures by 9 days post-infection, which look to have sporulated (Teng et al., 2025). While the progression of *E. tenella* parasites through these infective life stages *in vitro* represents a significant breakthrough to *Eimeria* research, it remains to be seen if the sporulated oocysts generated are infective when applied to a new batch of organoids (thereby permitting serial passaging *in vitro*) or, indeed, if they are infective *in vivo*.

Another example of a 3D cell culture model used to develop parasites through internal host life stages has recently been reported by Caygill et al. (2025). The authors showed that 3D HepG2/C3A hepatic spheroids co-cultured with red blood cells can be used to propagate *P. falciparum* parasites through multiple life stages. Liver spheroids initially infected with sporozoites were found to host schizonts and merozoites. Merozoites that egressed from the hepatic cells in the 3D spheroid subsequently invaded the co-cultured red blood cells. Prior to this, models for culturing liver stage *Plasmodium* parasites were considered relatively inaccessible due to their complexity and expensive cost. As well as providing an accessible template for future studies on liver stage parasites, the development of liver to blood stage parasites in a single setup represents a progressive step towards a system that permits the completion of the *Plasmodium* life cycle *in vitro*.

Organoids have also been explored for their utility in supporting the differentiation and maintenance of chronic parasite life stages. Standard methods for differentiating *T. gondii* into the chronic bradyzoite life stage from the acute tachyzoite life stage typically requires the addition of an external stimuli to drive differentiation, such as 0 % CO_2_ and alkali pH stress, Compound 1 (a trisubstituted pyrrole) and Compound 2 (an imidazopyridine) treatment, or IFN-gamma (IFN-γ). However, these artificial methods have always come with the caveat that they might not recapitulate cyst (bradyzoite) development to the extent realised *in vivo*. A key predilection site for chronic *T. gondii* infection is the brain. When Seo et al. (2020) added *T. gondii* to pluripotent stem cell-derived cerebral organoids, not only did tachyzoites invade and replicate within the cells of the 3D model, but the authors also claimed that the parasites innately differentiated into chronicstage bradyzoites without the addition of an external stimuli to drive differentiation. While the data on this is not particularly compelling due to a lack of upregulation in classic bradyzoite gene and protein markers following challenge, organoids were only challenged for less than three days. Infection with *T. gondii* induced an IFN-γ response in the cerebral organoids, as determined by transcriptional profiling of challenged vs non-challenged organoids, and hence it could be predicted that if parasites were maintained in this environment for longer, true bradyzoite cysts could eventually form. However, further evidence is required for this. Not all cells are created equally, and parasites can have a preference for particular cell types within a tissue, as alluded to above for *C. parvum* in intestinal organoids. Three days post-challenge with ME49 and RH strain *T. gondii* tachyzoites, parasites were found to preferentially co-localise with neuronal, astrocyte and oligodendrocyte cell types, whilst conversely being absent from radial glial cells contained within the cerebral organoids (Seo et al., 2020). Again, this demonstrates the applicability of employing a physiologically relevant culture model containing diverse cell types to better understand precisely how parasites interact with the host cellular environment in which they exist.

Similar to other protozoans, trypanosome research has been hindered by a lack of physiologically relevant *in vitro* models to investigate stages of this parasite, which spends considerable parts of its life in the host blood vessels. To address this gap, a recent study from Porqueddu et al. (2025) established 3D bovine brain and heart microvessel cultures as a model for *Trypanosoma congolense* infection. After 3 days in culture, both microvessel models displayed a tubular structure with a hollow lumen, with distinctly different morphologies. When endothelial cells in the model were exposed to two *T. congolense* strains at physiological flow rates, parasites were able to sequester to the endothelial cell surface in both models, with microfilaments extending to the parasite flagellum. However, differences were observed in the sequestration rates between the strains in the heart microvessel model, but not the brain model (Porqueddu et al., 2025). Not only is this study a major development for the *in vitro* modelling of trypanosome infection, but it also represents an effective demonstration of the tissue-specificity that these models exhibit and how this can improve understanding of host-parasite interactions across different parasite strains. It is easy to envisage how these and similar 3D blood vessel models could be applied across diverse vessel-dwelling parasites, such as *Plasmodium* spp. and *Schistosoma* spp.

#### 2.2.2 Studies on protozoan host-parasite interactions using organoids

Multicellular 2D and 3D culture has permitted unrivalled insights into protozoan host-parasite interactions that cannot otherwise be attained *in vivo*. For example, human brain organoids have been used to investigate the host cerebral response to *T. b. gambiense* (Chandrasegaran et al., 2023). This approach permitted studies on host gene expression response over time to establish an improved model to investigate the molecular basis of sleeping sickness caused by this kinetoplastid parasite. Infection with *T. b. gambiense* resulted in a mixed cell-mediated pro-inflammatory immune response characterised by the up-regulation of cytokines and chemokines related to type 1, 2 and 17 immune responses. Based on these results, more complex organoid models using co-culture with immune cells may help identify clinically relevant interactions between infected populations and immune cells, thereby better representing the broader network of host responses to parasite infection (Chandrasegaran et al., 2023).

The addition of immune-associated cell types has recently been supported by data from Lütkemeyer (2024), which showed that the immunological response to a parasite is different when organoids and parasites are cultured alone or with monocytes, demonstrating the additional information that can be gained from organoid-immune cell co-culture. For instance, the immunological response markers *TNF-α, IFN-α/β/γ, IL-6, IL-10, IL-12p70, CCL2, CCL3* and *GM-CSF* were considerably up-regulated only when all three components; the kinetoplastid *Leishmania infantum*, organoids (hepatic) and monocytes, were co-cultured in a single system. Moreover, responses were limited in **embedded cultures** compared to **suspension cultures** (Fig. 1). Despite these findings, it is also important to note that a more complex co-culture system is not always necessary to induce host responses by a protozoan parasite. For instance, Ashander et al. (2025) found that *IL-6, CXCL8, CXCL10* and *CCL2* were all up-regulated in retinal organoids challenged with live *T. gondii* parasites compared to unchallenged controls. However, *IL-6, CXCL8* and *CXCL10* were also up-regulated in organoids treated with heat-killed parasites in the same experiment, although *CXCL10* and *IL-6* expression reduced to baseline over the course of seven days. Taken together, this supports the notion that careful consideration is required when developing organoid systems for modelling host responses to parasite infection *in vitro*.

#### 2.2.3 Models to study host interactions with parasite-derived molecules

An alternative approach to modelling host responses to live parasites is to model aspects of diseases − including interactions with parasite products (e.g. proteins, lipids, small RNAs) − associated with infection, rather than directly applying live parasites into the model themselves. This approach was taken by Harbuzariu et al. (2019) to study heme-mediated brain injury, a pathology associated with cerebral malaria. Heme treatment of human iPSC-derived brain cortical organoids was shown to induce early apoptosis and necrosis, which was associated with increases in the pro-inflammatory chemokine CXCL-10 and its receptor CXCR3. In another example, exposure of 3D microvessel models to *P. falciparum* products resulted in a breakdown in endothelial structure, including reduced microvilli-like projections and loss of endothelial junction markers (Korbmacher et al., 2025). Collectively, these studies represent effective demonstrations of how 3D cell culture models are being applied to investigate pathology associated with cerebral malaria.

Some secreted parasite products are encapsulated within extracellular vesicles (EVs). While EVs have been a key focus of 3D experimental models of host-parasite interactions for helminths (see below), this has not been the case for protozoan research in 3D cell culture models to date, with very few publications on this topic. This has perhaps been limited by inadequate methodologies to robustly isolate secreted products and EVs from protozoan parasites, since many are obligatory intracellular parasites. A recent study from Cruz-Bustos et al. (2025) set a precedent by reporting the isolation and proteomic characterization of EVs from *T. gondii* parasites cultured in various cell types. Moreover, a study involving the treatment of intestinal Caco-2 cells with EVs from *G. intestinalis* revealed changes in host cell gene expression following treatment (Yang et al., 2024), demonstrating the capacity for protozoan EVs and their cargo to manipulate host cell gene expression. The isolation of secreted material from protozoan parasites will further broaden the application of organoids and 3D cell culture models to facilitate investigations into how parasite molecules interact with host cells in a robust surrogate of *in vivo* tissue.

#### 2.2.4 Protozoa as delivery systems

Beyond their role as infectious agents, protozoa have also been co-opted as innovative biotherapeutic tools, with recent research exploring their potential as living delivery vehicles to transport host-encoded proteins into otherwise inaccessible tissues. One such novel and unconventional use of protozoa-organoid systems was demonstrated by Bracha et al. (2024). The authors showed that genetically modified *T. gondii* could act as a surrogate delivery system to deliver exogenous therapeutic host protein to the brain (in cultured cells, brain organoids, and *in vivo*) in cases where a deficiency leads to pathology (e.g. Rett syndrome). This exploited the parasite’s ability to traverse the blood–brain barrier and the relative ease with which the parasite can be genetically modified. Prior to moving into a live animal model, the authors demonstrated that GRA16-fused MeCP2 (a host transcription factor) could be deposited by modified parasites into neuronal cells within human cortical brain organoids. Moreover, organoids infected with *T. gondii* expressing GRA16-fused MeCP2 exhibited a different gene expression profile to organoids infected with parental control parasites 50 days post-infection, characterised by upregulation of the “Reactome transcriptional regulation by MECP2” pathway. This coincided with increased *CREB1* expression (a MECP2-interacting transcription factor) and decreased *MEF2C* expression (a gene known to be inhibited by MECP2). Collectively, this indicated that the delivered transcription factor was functional in host neuronal cells within the organoids, justifying further investigation *in vivo*.

The range of tissue types, hosts and protozoan species modelled in 3D cell culture systems to date, as well as the diverse experimental approaches undertaken, is a testament to the accessibility and applicability of this technology for modelling protozoan infection *in vitro*. However, key considerations should be applied in the design of such models. For instance, the growth factors and inhibitors used to stimulate the growth of different organoids could affect the performance of parasites in culture. As an example, rho-associated protein kinase (ROCK) inhibitor is used in the culture of various organoid types to promote cell survival and block apoptosis, however the inclusion of this inhibitor in intestinal organoid cultures can block replication of *T. gondii* tachyzoites, with invaded intracellular parasites remaining as a single cell within the parasitophorous vacuole up to 11 days post-infection (D. Smith, *pers obs*). As mentioned above, the design of the experiment, for example organoid orientation (cellular polarity), the complexity of cells present, and whether organoids are embedded in a matrix or are in suspension, has the potential to greatly influence the results generated and indicates the need for well-characterised models from the outset (Fig. 1).

### 2.3 Multicellular 2D and 3D cell culture systems to study helminth infections

Helminths are a diverse group of parasites that vary in size, morphology, and life cycle strategies, encompassing species from two major phyla, Nematoda (roundworms) and Platyhelminths (flatworms). Routes of infection and tissue tropism vary substantially between species. Some, like whipworms, infect via the oral route and remain confined to the GI tract, interacting primarily with the epithelial barrier. Others, including skin-penetrating parasitic nematodes, such as *Strongyloides* spp., or systemically migrating trematodes and cestodes, such as *Fasciola hepatica* and *Echinococcus* spp., traverse multiple tissue types en-route to their target niche, which may include the liver, lungs, or muscle. These varied strategies, along with parasite developmental stages ranging from microscopic larvae to metres-long adults, influence how helminths interact with host cells and evade immune responses.

When designing multicellular 2D and 3D *in vitro* models to study helminth infections, it is important to consider the stage, size, motility, and target tissue of the helminth of interest. While not all stages are amenable to *in vitro* culture due to their size or complexity, this diversity presents an opportunity to carefully tailor experimental systems to mimic key aspects of the *in vivo* environment. By recreating the appropriate structural, biochemical and cellular context, these advanced models can generate mechanistic insights into host-parasite interactions that are often difficult or impossible to capture *in vivo*. As such, multicellular 2D and 3D culture systems represent a powerful platform for studying helminth biology, offering an alternative to conventional 2D monolayers or *in vivo* animal models in a setting that recapitulates key physiological features.

In this section, we summarise the most relevant results in the helminthology field to date using multicellular 2D and 3D culture models to study helminth invasion and the effects of parasite-derived factors, including microRNAs and EVs, in modulating epithelial responses, stem cell niches, and innate immune signalling (Table 2).

#### 2.3.1 Impacts of nematode extracellular vesicles (EVs) on epithelial cultures

The first documented application of organoid technology in helminth research examined the uptake of nematode EVs derived from *Trichuris muris* and *Nippostrongylus brasiliensis* by murine 3D intestinal organoids (Eichenberger et al., 2018a; Eichenberger et al., 2018b). EVs were microinjected into the organoid lumen, and their uptake was shown to be energy-dependent, as internalisation was significantly reduced at 4 °C whereby cellular metabolism would be presumably lower. Responses to intestinal epithelial cells were later studied by Duque-Correa et al. (2020) who, using RNA sequencing, demonstrated that microinjection of adult *T. muris* EVs into murine 3D caecal organoids (**caecaloids**) resulted in downregulation of interferon-stimulated genes, suggesting an anti-inflammatory effect on the host epithelium.

Organoid polarisation affects epithelial responses to nematode EVs. White et al. (2024) used a 2D murine SI organoid (**enteroid**) model to investigate how *Heligmosomoides polygyrus bakeri* L4, adult worms, and their EVs interact with intestinal epithelial cells. They observed that host transcriptional responses varied significantly depending on whether adult worms were applied to the apical or basal surface, with stronger responses, particularly in genes associated with interferon signalling and immune regulation, elicited from basal exposure (Fig. 1). This suggests that receptor localisation and cellular polarity are critical factors shaping host sensing of nematode stimuli. While no differentially expressed genes were detected in bulk RNA-seq following apical exposure to *H. polygyrus bakeri* EVs, transcriptomic changes were revealed in sorted epithelial subpopulations, including tuft and goblet cells. It remains to be seen whether basal treatment with EVs would elicit broader or more robust changes in host gene expression, as observed with adult worm exposure. When Bellini et al. (2024) used 2D human colonic organoids to investigate the effects of *Anisakis* spp. EVs, they noted altered expression of genes involved in antigen presentation, connective tissue biogenesis, and cell fate regulation, including downregulation of *EPHB2*, a key component of TGFb–PDGF signalling, suggesting modulation of host immune responses. Comparative studies examining trematode EV impacts on organoid responses remain limited, but this represents an exciting area for further investigation using complementary approaches such as advanced imaging, proteomics, and co-culture systems incorporating immune or stromal cells.

#### 2.3.2 Effects of other nematode excretory-secretory (ES) products on 3D organoids

In addition to studying the impacts of nematode EVs on host cells, GI organoids have also been used to study the effects of nematodes and their other excretory-secretory (ES) products, the contents of which are differentially produced by various parasite life stages to facilitate host invasion and establishment on the intestinal epithelia. For example, *in vivo* infection with the murine nematode *H. polygyrus bakeri* results in a foetal-like reversion of the host intestinal stem cell niche during establishment, characterised by crypt hyperproliferation and morphological changes associated with granuloma formation and IFN-γ signalling (Nusse et al., 2018). SI organoids have been instrumental in advancing our understanding of this effect: the culture of crypt cells isolated from *H. polygyrus bakeri* infected mice has been shown to present a **“cystic” morphology**, lacking the characteristic crypt budding and cell differentiation of typical SI organoids and thus indicating their undifferentiated state. Moreover, murine SI organoids treated with adult worm ES exhibit transcriptional changes associated with stemness and reduced differentiation (Nusse et al., 2018; Drurey et al., 2021; Karo-Atar et al., 2022). It is important to note that in their study, the researchers referred to these cystic organoids as “spheroids”, however in this review the term spheroid is used to refer to a specific type of 3D cell culture model (see Glossary). Following extraction of crypts from *H. polygyrus bakeri*-infected mice at different time points post-infection, Karo-Atar et al. (2022) observed that organoids grown from the crypts yielded typical budding morphology at early time points (inc. day 6), but crypts collected at day 14 post-infection adopted the cystic morphology. In contrast, Nusse et al. (2018) observed a cystic phenotype in organoids derived from day-6 infected mice. A key difference between these two studies is that Nusse et al. (2018) enriched for *H. polygyrus bakeri*-exposed/granuloma-associated primary crypt cells (using a fluorescent Sca-1 reporter) prior to setting up organoid cultures, whereas Karo-Atar et al. (2022) established organoid cultures from bulk intestinal crypts. Without the enrichment for parasite-exposed stem cells, this might explain why the phenotype was not observable as early in the Karo-Atar study. Collectively, these studies exemplify the application of organoid technology as a readout of the effects of *in vivo* infection to dramatically alter the stem cell niche as *ex vivo* growth of organoids retain a blueprint of the individual tissue that they are derived from. Additionally, this finding highlights the importance of sourcing suitable donors for generation of organoids from primary cells, particularly when working with material derived from species other than laboratory rodents, which can be parasite-exposed prior to sample collection.

Drurey et al. (2021) further showed that *H. polygyrus bakeri* ES products suppress SI epithelial organoid differentiation into tuft and goblet cells, secretory cell types required for nematode clearance from the SI (Gerbe et al., 2016). Perez et al. (2025b) found that organoids acquired a cystic phenotype following treatment with concentrated ES from the adult stage of the ovine nematode *Hae-monchus contortus*, but not with unconcentrated ES, indicating a dose-dependent effect. These data indicate that GI nematodes can manipulate tissue architecture to favour their survival and this can be mimicked in organoids following incubation with parasite-derived ES products (Drurey et al., 2021; Karo-Atar et al., 2022).

#### 2.3.3 Effect of nematode microRNAs on GI organoids

MicroRNAs (miRNAs) are small regulatory RNAs that modulate gene-expression post-transcriptionally by binding to the 3′ UTR of target mRNAs, leading to inhibition of protein translation and transcript degradation (Chekulaeva and Filipowicz, 2009). Several groups have shown that helminths secrete miRNAs and these may modulate host gene expression (Buck et al., 2014; Chowdhury et al., 2024a; Chowdhury et al., 2024b). Effects have been studied using cell lines but not organoids. In a previous study, Gu et al. (2017) focused on the function of a secreted miRNA (miR-5352) conserved in GI nematodes but not found in filarial nematodes. The evolutionary conservation of this miRNA in nematodes infecting a given tissue niche could mean that it had a particular function at that site, in this case to modulate host gene expression in the GI mucosa. Using ovine gastric and SI organoids, Pérez et al. (2025b) successfully delivered a miR-5352 mimic into organoid cells and monitored associated changes in epithelial cell phenotype and gene expression. Delivery was achieved using a lipid-based transfection reagent (DharmaFECT™), highlighting the feasibility of miRNA manipulation in ruminant organoid systems. It was observed that miR-5352 suppressed IL-13-induced differentiation of GI epithelial secretory cells, including tuft and goblet cells, similar to the effect of *H. polygyrus* or *H. contortus* complete ES (Drurey et al., 2021; Perez et al., 2025b). Simultaneously, miR-5352 maintained organoid stemness, modulating Wnt and Notch signalling pathways (Perez et al., 2025b). The Kruppel-like transcription factor KLF-4, a key promoter of cellular differentiation that normally suppresses Wnt signalling, was identified as a critical target; *klf-4* expression was significantly reduced in miR-5352-transfected organoids (Perez et al., 2025b). Notably, nematode miR-5352 shares its seed sequence with mammalian miR-92a, also known to suppress *klf-4* (Lv et al., 2016*)*, suggesting that GI nematodes may have hijacked a host regulatory pathway to attenuate innate immune responses and enhance their survival.

#### 2.3.4 Modelling nematode infection in 2D and 3D organoids

One of the most exciting applications of organoid models is that both 3D and 2D models can be used to investigate host-parasite interactions with live worms. Tissue mechanisms of invasion by live GI nematodes are relatively poorly understood. For example, L3 stage larvae of the ruminant gastric stomach worms *Ostertagia ostertagi* and *Teladorsagia circumcincta* enter the gastric glands, where they moult to later larval stages, before emerging from glands as adults that reside in the gastric lumen, causing significant pathology to their hosts. However, there is little understanding of the specific host-parasite interactions and invasion processes that take place to permit infection and subsequent emergence.

The first application of these nematodes on 3D organoid cultures was demonstrated for *T. circumcincta*, with L3 larvae found to penetrate the epithelium as early as two hours post-incubation and take up residence within the lumen organoids, suggesting the feasibility of organoids as minimally invasive infection models (Smith et al., 2021). Similarly, *O. ostertagi* larvae displayed active probing and invasion behaviours, leading to striking morphological alterations (“ballooning” or swelling) in abomasal organoids (Faber et al., 2022). The larvae did not develop beyond L3 stage but were active and survived for several weeks. How these parasites invade such a small space, such as the gastric gland, is beginning to be appreciated. In both studies on the ruminant stomach worms, the authors observed a “ballooning”-like phenotype in organoids that contained live L3 stage larvae (Smith et al., 2021; Faber et al., 2022). It is important to note that this is distinctly different to the cystic de-differentiation phenotype associated with other nematodes described above, as the effect here was observable within 30–60 min in ruminant gastric organoids treated with ES products, indicating a shift in osmotic balance between the organoid luminal space and the surrounding culture media in treated organoids (Faber et al., 2022). Upon observing L3 larvae display penetrative behaviour and migration across the gastric epithelium in an organoid model, the authors were encouraged to see if there was evidence of this behaviour *in vivo*. This led to the revision of literature that show instances in which larvae could be seen situated beneath the gastric epithelium in tissue sections from infected animals (Thomas and Probert, 1993). It will be interesting to examine if GI organoids can help identify triggers for larval development and emergence and how these processes may be inhibited by drugs or vaccination.

Duque-Correa et al. (2022) pioneered a *T. muris* infection model using 2D murine caecaloids to replicate invasion of the caecal epithelia by first-stage (L1) whipworm larvae. Using 2D caecaloids grown in transwells, larvae interacted with the apical epithelial surface, mirroring their natural infection route. The caecaloid model successfully recapitulated key features of early *T. muris* infection, including mucus degradation, epithelial penetration, and syncytial tunnel formation. Subsequently, a novel transgel organoid system has helped refine studies on *T. muris* interactions by providing a membrane-free, geometrically structured platform for independent manipulation of epithelial polarity, enhancing live imaging and long-term infection studies (Hofer et al., 2025). The system combines features of classical transwell systems and hydrogel scaffolds, allowing visualisation of *T. muris* L1 larval invasion in real-time, and revealing that motility alone does not determine infection success, but that initial curling behaviour may facilitate penetration. These *in vitro* models of *T. muris* infection support the growth and moulting of whipworms (M. Duque-Correa, *pers obs*). Multicellular 2D monolayers are likely to facilitate the development of other nematode larvae, providing much needed *in vitro* culture systems to progress our understanding of nematode biology, with potential for use as screening platforms for functional genomics and novel therapeutics (see later).

Expanding to other nematode species, Hellman et al. (2024) explored interactions between live *Parascaris univalens* cyathostominae and *Strongylus vulgaris* larvae and the equine intestinal epithelium using equine SI organoid-derived 2D monolayers. Stimulation with type-2 cytokines IL-4/IL-13 promotes epithelial differentiation into tuft and goblet cells. The expansion of these cell types in response to particular nematode infections *in vivo* has typically been considered to be part of a positive feedback loop involving a relay of IL-25 signalling from tuft cells to innate lymphoid cells (ILC2s), which in turn release IL-4 and IL-13 to stimulate tuft and goblet cell hyperplasia (von Moltke et al., 2016). What is particularly notable here is that co-culture with *P. univalens* further enhanced *MUC2* expression, a marker for goblet cells, indicating parasite-driven modulation of goblet cell expansion and mucus production. Since IL-4 and IL-13 concentrations were static across conditions, this suggests an independent parasite-associated factor can also drive the expansion of secretory lineage epithelial cells, independent of ILC2 signalling molecules (Hellman et al., 2024). Live-cell imaging also revealed epithelial morphological changes in response to larval exposure, independent of cytokine stimulation. These findings contrast with the de-differentiation effect observed in organoids in response to other nematode parasite species and warrant further investigation.

#### 2.3.5 Modelling platyhelminth infections using 3D cell culture systems

Chronic clonorchiasis is a long-term disease caused by infection with the carcinogenic human liver fluke *Clonorchis sinensis* that resides in the bile ducts of the liver of definitive hosts where it causes persistent irritation and inflammation. While many individuals with clonorchiasis remain asymptomatic, chronic infections can lead to significant hepatobiliary complications including cholangiocarcinoma. Kim et al. (2021) showed 3D human cholangiocyte spheroids model aspects of chronic clonorchiasis. Upon exposure to ES products from *C. sinensis*, the spheroids underwent significant transcriptomic changes primarily related to immune responses and ECM remodelling, mirroring the inflammatory and fibrotic alterations seen in chronic liver fluke infections.

The use of spheroids in modelling platyhelminth infections is not limited to simply gaining a better understanding of mammalian host responses to these parasites. HepG2 spheroids have also been used to model parasite life stage development. Studying the infective stages of helminth parasites *in vitro* has long been constrained by the limited survival and developmental progression of parasites outside of their mammalian hosts. This is particularly true for *F. hepatica* newly excysted juveniles (NEJ), which rapidly invade and damage hepatic tissue, and thus remain inaccessible for direct *in vivo* observation due to their small size relative and tissue location. Unlike many protozoan parasites, *F. hepatica* and most other parasitic helminths are not amenable to transgenesis, making them unsuitable for the development of fluorescent reporter lines for *in vivo* tracking (Quinzo et al., 2022). To address this challenge for *F. hepatica*, Vitkauskaite et al. (2025) developed a tractable 3D culture system using HepG2 spheroids that mimic the hepatic microenvironment to an extent that facilitates the sustained co-culture of NEJs for over 21 days. Parasites exhibited significantly enhanced survival, growth and development, were observed to actively graze on spheroid peripheries within 24 h post excystment, and excreted/secreted temporally regulated cysteine proteases (FhCL3 and FhCL1/2) in patterns that resemble *in vivo* kinetics. Co-culture with HepG2 spheroids promoted marked tegumental and somatic development, including the elaboration of spines, oral and ventral sucker papillae, and development of the bifurcated gut and associated musculature, hallmarks of parasite maturation. This study together with the previously mentioned use of HepG2 spheroids to replicate *P. falciparum* propagation (Caygill et al., 2025) demonstrate the utility of 3D cell culture systems to support parasite life stage progression *in vitro*. Moreover, akin to recent applications of organoid technology for modelling infection by nematode larvae, this spheroid-based system provides a crucial window into our understanding of the liver fluke infection, namely the **‘early infection gap’**, the period between NEJ penetration of the host SI and maturity into ~21-day-old juveniles in the liver parenchyma. Until now, research has largely focused on excysted NEJ cultured in simple liquid media, invasion across explant mice intestines, or later-stage liver migrants, neglecting the most clinically relevant phase of infection, when pathology is most severe and interventions such as vaccines and drugs are designed to act (Lalor et al., 2021). The Vitkauskaite et al. (2025) model is the first to support continuous observation of liver fluke development across this gap under controlled conditions *in vitro*.

As with any model, biological relevance must be validated against *in vivo* parasites. Here, advances in single-cell and spatial transcriptomics offer new avenues for comparison. Gramberg et al. (2024) recently provided a spatial gene expression atlas of *F. hepatica* adult tissues, revealing distinct transcriptional signatures across eight tissue types, including the tegument and gut, two sites crucial to host-parasite interaction and drug/vaccine target discovery. The extension of such omics-based profiling to juvenile stages, particularly in parallel with spheroid-grown NEJ, would enable rigorous benchmarking of gene expression, cellular identity, and developmental trajectories. Single-cell and spatial approaches could help determine whether NEJ developing on spheroids (or parasites grown in any other co-culture system) faithfully recapitulate the transcriptional identity of migrating juveniles within the liver parenchyma, while also identifying key stimuli lacking in the current model.

Hepatic 3D culture models have also been applied to study *Echinococcus multilocularis* larval development (Li et al., 2018). Co-culture of hepatocytes and mesenchymal stem cells on a collagen scaffold preserved hepatocyte functionality, including albumin secretion, urea synthesis, and cytochrome P450 enzyme activity for up to 28 days. Notably, 3D-cultured hepatocytes facilitated the rapid de-differentiation of *E. multilocularis* protoscoleces into infective vesicles within eight weeks, effectively recapitulating *in vivo* conditions. For most parasitic helminths, *in vitro* development and maintenance of mammalian stages are major challenges to studying parasite biology and developing robust functional genomics or drug screening platforms. The advances being made in multicellular 2D and 3D cell/helminth co-culture models have significant potential to transform the field of helminthology research.

## 3 Lesson 2: Present challenges and limitations in the uptake of multicellular 2D and 3D cell technologies

While multicellular 2D and 3D cell culture systems offer powerful platforms to study host–parasite interactions, their broader adoption in parasitology has been limited by technical, biological, and logistical challenges. These models require a careful balance between physiological relevance and experimental tractability. Complexities around reproducibility, tissue-specific growth conditions, and accessibility across different host species hinder widespread uptake, particularly in veterinary and non-model animal systems. This section explores three critical barriers that currently constrain the field: reproducibility and standardisation, physical limitations related to tissue viability and structure, and the accessibility of models from diverse host species. Recognising and addressing these challenges is essential to unlocking the full potential of advanced culture platforms in parasitology research.

### 3.1 Reproducibility and standardisation of culture systems

One of the most frequently cited limitations of spheroid and organoid research is the lack of standardisation across laboratories (Zhao et al., 2022). Variability in protocols for tissue dissociation, matrix embedding, and growth factor supplementation often results in models with inconsistent architecture, cell composition, and function. Growth media can be prepared in-house or purchased from different suppliers, with inherent batch-to-batch variation in quality, and undefined components such as sera and Matrigel^®^ contributing further to batch-specific effects. These inconsistencies can impact organoid transcriptomic profiles, cellular behaviour, and susceptibility to infection or drug treatment, affecting reproducibility across research groups (Zhao et al., 2022).

Moreover, long-term passaging of organoids may result in genetic drift, epigenetic changes, and phenotypic divergence from the original tissue source, a consideration shared with the sustained culture of traditional immortalised 2D cell lines (Chehelgerdi et al., 2023). Without rigorous benchmarking and −omics-based validation, such drift can undermine the reliability of mechanistic or comparative studies. In the broader organoid field, calls have been made to adopt generalised standards for cell isolation, seeding density, media composition, and data deposition to support model reproducibility across studies. To enhance the reliability of organoid-based research, future efforts should ensure standardised methods are followed, including the use of defined and consistent media compositions and robust molecular characterisation of the cellular diversity and expression in organoids with comparison to the source material from which they were derived.

### 3.2 Oxygenation, necrosis, and size limitations in 3D systems

Advanced 3D cell culture systems offer greater tissue complexity than monolayers but face size-dependent limitations in nutrient and gas exchange (Huang et al., 2022). As spheroids increase in size, diffusion barriers lead to steep oxygen and nutrient gradients, resulting in localised hypoxia, waste accumulation, and ultimately cell death. This often gives rise to necrotic centres, particularly in spheroids exceeding 500 μm in diameter, which can compromise tissue architecture and function. In contrast, epithelial organoids, such as those derived from the gastrointestinal tract, typically maintain a central lumen and shed senescent or apoptotic cells into this space, partially mitigating the impacts of necrosis. Nonetheless, both spheroids and organoids remain constrained by diffusion limits that reduce physiological fidelity over time, especially during long-term experiments or co-culture with parasites.

To address these issues, dynamic culture systems such as bioreactors and microfluidic systems (e.g. organismoids) have been developed to enhance nutrient and oxygen delivery, facilitate waste removal, and extend the viability of larger constructs (Velasco et al., 2020; Hetzel et al., 2023; Licata et al., 2023; Zhu et al., 2023; Ye et al., 2024). In some cases, smaller spheroid sizes or embedding within perfused matrices can mitigate necrosis, though these solutions come with their own technical constraints and trade-offs. The Vitkauskaite et al. (2025) *F. hepatica* co-culture model overcomes some of these limitations by plating individual spheroids in triplet overnight, allowing them to knit together on the periphery before the addition of parasites to the wells, thus expanding the available surface area for host-parasite engagement, which is critical as they grow, while maintaining a reduced tissue volume that supports adequate nutrient and oxygen exchange. Emerging scaffolds that promote vascularisation within microtissues also show promise in prolonging construct viability and mimicking *in vivo* conditions more closely (Liu et al., 2023). Ultimately, careful consideration of tissue type, parasite size and growth dynamics, culture duration, oxygenation strategy, and system design is essential to ensure model stability and biological relevance − particularly for long-term co-culture or drug screening applications.

### 3.3 Species representation and model accessibility

Despite current limitations, 3D cell culture technology has provided unprecedented opportunities to gain novel insights into host-parasite interactions *in vitro*. To fully realise their potential, especially in veterinary parasitology, broader access to models derived from non-traditional hosts, such as livestock and aquatic species, is urgently needed. Currently, these models must be generated *de novo* from primary tissues, limiting their scalability, reproducibility and uptake across research groups. While commercially available spheroid and organoid systems have begun to emerge for certain human and murine tissues, equivalent models for veterinary species remain scarce. The development and commercialisation of well-characterised 3D models from diverse tissues and species, coupled with the creation of curated biobanks covering a range of tissues and hosts, would represent a critical step forward. These resources would facilitate standardisation, reduce barriers to entry, and support collaborative research across institutions and disciplines. By addressing these bottlenecks and continuing to refine culture systems, multicellular 2D and 3D models will become increasingly powerful tools for studying parasite biology, modelling infection, and developing more targeted therapeutic strategies.

## 4 Lesson 3: Priority areas and future applications of 3D models to the study of parasitology

As multicellular 2D and 3D cell culture technologies continue to mature, they offer new opportunities to ask more sophisticated biological questions and address long-standing challenges in parasitology. These platforms are not only reshaping how we model parasite infections and tissue responses but also have the potential to enhance drug discovery pipelines, improve our understanding of host specificity and resistance, and reduce reliance on animal experimentation. Organoids and multicellular 2D culture systems derived from individual human and animal donors hold particular promise for uncovering why certain individuals or breeds respond differently to infection. In human models, this personalised approach can facilitate the identification of patient-specific risk factors and targeted therapeutic strategies (Bose et al., 2021). In livestock, different species and breeds exhibit varying levels of resistance, and primary organoids offer a controlled system to study the genetic and physiological factors influencing these differences. This knowledge may one day support selective breeding programs by identifying animals with superior resistance at a cellular level. Alternatively, 3D models can be utilised to progress our understanding of effective host resistance mechanisms. Using these models to identify specific parasite antigens that have a key functional role in interacting with host tissue will help to underpin future vaccine development, ultimately reducing the need for chemical deworming and, hopefully, slowing the development of drug resistance.

### 4.1 Organoids as a tool for drug discovery

#### 4.1.1 Applications in hepatotoxicity and drug metabolism

Drug discovery and development pipelines across all disease areas are increasingly integrating advanced *in vitro* systems to improve the predictiveness and safety of preclinical testing (Harrison et al., 2021; Shinozawa et al., 2021; Arora et al., 2023; Bouwmeester et al., 2023; Hendriks et al., 2023; Yang et al., 2023a; Meyer et al., 2024). A major challenge for pharmaceutical development is drug-induced liver injury (DILI), which remains one of the leading causes of drug attrition, with an estimated 32 % of novel pharmaceuticals removed from the market due to liver toxicity (Downing et al., 2017). This has driven the development of more functionally representative models to assess early-stage toxicity and pharmacokinetics, particularly liver-based models that more accurately recapitulate human hepatic function than traditional immortalised 2D cell lines (Meyer et al., 2024).

In this context, hepatic spheroids and organoids have emerged as particularly powerful and flexible tools, offering improved metabolic function, bile production, and tissue architecture compared to traditional models. Commercial platforms such as Gibco™ Human Spheroid-Qualified Hepatocytes, derived from single donors, exhibit both phase I and II enzyme activity, albumin production, and the ability to form bile canaliculi, making them well-suited to study liver metabolism and toxicity (ThermoFisher, 2025). These features make 3D liver models especially useful for evaluating hepatotoxicity, drug metabolism, and therapeutic index across a range of compound classes, including anti-parasitic agents.

For parasitology, such platforms are increasingly important. They provide accurate models of native tissues for assessing host responses to parasite infection or exposure to parasite-derived products, particularly in hepatic systems affected by parasites such as *F. hepatica, Plasmodium* spp., or *Echinococcus* spp. Additionally, these systems can support the early screening and refinement of candidate compounds where host-specific metabolism or hepatotoxicity may otherwise obscure antiparasitic efficacy.

Intrahepatic cholangiocyte organoids derived during primary human liver biopsy were analysed for their gene expression of phase I and II enzymes involved in drug metabolism, such as CYP450 genes, albumin, and hepatic transporters upon differentiation (Bouwmeester et al., 2023). When challenged with known hepatotoxic drugs such as diclofenac, perhexiline, and troglitazone alongside primary hepatocyte cultures and HepaRG cells, the organoids produced comparable EC50 values (Bouwmeester et al., 2023). Similarly, Shinozawa et al. (2021) tested IiPSC-derived hepatic organoids against 238 currently marketed drugs at four different concentrations to investigate their cell viability and mitochondrial or cholestatic toxicity *in vitro*. This high-throughput liver organoid toxicity screening identified cholestatic stress as a driver of liver damage, as opposed to mitochondrial stress. Hepatic organoids derived from hPSCs co-cultured with hepatic stellate cells have also been used to predict DILI. Shin et al. (2025) assessed the ability of these cultures to predict liver injury using 12 known hepatotoxic reference compounds. When challenged, the organoids displayed similar cytotoxicity and increased inflammation as previously reported *in vivo*, validating the use of these models in early-stage drug development to predict potential damage downstream (Shin et al., 2025).

#### 4.1.2 Host-parasite response profiling and stage-specific drug evaluation

Recent advances now extend these tools to parasite co-culture models, permitting observation of previously inaccessible phenotypes. The *F. hepatica* NEJ co-culture system developed by Vitkauskaite et al. (2025) sets the groundwork for the establishment of a bioassay for phenotype-based drug screening. The current model enables observation of dynamic traits and behaviours such as NEJ feeding, digestion, tissue invasion, and cysteine protease secretion, processes that can be visualised in real time and quantified using live-cell imaging and other advanced microscopy or biochemical techniques to form the basis of behavioural ethograms. The addition of transcriptomic and proteomic profiling will further define parasite and host responses to treatment in the presence of drugs, provided that the “normal” baseline phenotype is first well-characterised. This approach has particular relevance for the study of *F. hepatica*, where drug efficacy is known to vary across life cycle stages (Fairweather et al., 2020). Triclabendazole, for example, is the only registered drug with high efficacy (>90 %) against both immature and adult flukes, yet the specific mechanism of action of the active metabolites, triclabendazole sulfoxide and triclabendazole sulfone, remain unknown. A 3D liver–based drug metabolism bioassay offers a unique opportunity to expedite screening of current and candidate compounds against the immature migratory liver stage prior to embarking on costly *in vivo* studies, thereby streamlining discovery and enabling prioritisation of the most promising molecules. Moreover, the translatability and applicability of these approaches extend beyond *Fasciola* and have the potential to advance drug development across parasitology. Due to the financial and ethical implications involved in large-scale animal and human studies, organoids represent a viable alternative for early-stage drug candidate testing and screening of metabolism, bioavailability (including in host-parasite co-culture models) and tissue toxicity. In addition, the ability to derive organoids from a variety of species, such as cattle, sheep, and goats, highlights the potential for species-specific drug testing in economically important livestock hosts. This would help identify and account for species differences in response to medications at an early stage, reducing the need for animal trials. Alongside liver organoids, this technology could also be applied to assess the toxicity of novel drugs on specific tissues. As an example, cardiac organoids have been used to assess the impact of small molecules on cardiac function, specifically noting disparities between the response of organoids and 2D cell lines due to the organoids’ increased complexity (Li et al., 2022).

When applied towards the study of anti-parasitic agents, Melcón-Fernandez et al. (2024) evaluated the impact of 1,5- and 1,8-substituted fused naphthyridines for their anti-leishmanial effects. To assess compound toxicity following oral administration, murine intestinal organoids were challenged with each molecule and screened for drug tolerance, with the results compared to their anti-leishmanial impact. Similarly, these organoids were used to assess the cytotoxicity and efficacy of a combination of clinically available leishmanicidal drugs (Melcón-Fernández et al., 2024). These studies illustrate how organoid-based systems can be used not only to assess compound efficacy, but also to simultaneously evaluate host toxicity, providing a dual-screening approach that is highly relevant to antiparasitic drug discovery.

Ultimately, the expansion of 3D parasite co-culture systems involving either intracellular protozoa or helminths offers an important step forward in understanding how both host and parasite respond to treatment. Taken together, organoids and spheroids represent a scalable and ethically sound platform for early stage antiparasitic drug screening. Their application across diverse parasite groups and host tissues, coupled with the ability to incorporate genetic manipulation, co-culture with parasites, and produce a range of functional readouts, makes them ideally suited to address the growing challenges of drug resistance and limited therapeutic innovation in parasitology.

### 4.2 Vaccine modelling using 3D culture

Vaccines are vital tools in reducing pathogen-related morbidity and mortality. By introducing pathogen-specific antigens, vaccines elicit a protective immune response aimed at preventing or limiting infection or disease (Vetter et al., 2018). However, there are relatively few anti-parasite vaccines commercially available and there is an important biotechnological gap to meet global demand for novel parasite control strategies. Together with the selection of antigens, adjuvants that enhance the desired immunogenic response, and the vaccine delivery system of choice, are both important aspects of vaccine design. Delivery routes include intradermal, subcutaneous and intramuscular injection as well as mucosal routes, such as intranasal and oral delivery (Bouazzaoui and Abdellatif, 2024). The use of one delivery system over another depends on which pathogen the vaccine is targeting. For example, a mucosal vaccine delivery would be better suited against respiratory viruses and enteric infections by inducing immunity at the sites of infection.

#### 4.2.1 Investigating mucosal antigen uptake and immunogenicity

Traditional vaccine development currently relies on a combination of animal models that are expensive, time- and resource-intensive, and raise ethical issues, and traditional 2D cell culture systems that often fail to fully replicate the complexities of immune responses and could be incompatible with the target species. Organoids represent novel platforms particularly suited to investigate the uptake of antigens at the mucosal surface, which is an important site for a range of protozoan and helminth parasites. In addition, they also have the potential to evaluate responses to prototype adjuvants and delivery systems (see below). The use of organoids to support the development of vaccines against parasites is especially pertinent in light of a recent Global Veterinary Vaccinology Research and Innovation Landscape Survey Report commissioned by the EU-supported STAR-IDAZ International Research Consortium on Animal Health, which highlights mucosal vaccines, and our fundamental understanding of the host immunological responses to them, as major technological and knowledge gaps in the prevention of infectious diseases (STAR-IDAZ, 2022).

#### 4.2.2 Adaptive immune responses in immune–organoid platforms

Although the use of organoids remains unexplored for the development of vaccines against parasites, examples where this technology has been exploited for other pathogens are outlined below. In 2021, Wagar et al., 2021 used human tonsil-derived immune organoids to study the adaptive immune response to live-attenuated influenza vaccine (LAIV). Upon stimulation with LAIV, the tonsil organoids displayed significant B cell maturation along with the production of influenza-specific IgG antibodies when compared to unstimulated organoids. It is feasible to consider that a similar approach could be used to identify novel anti-parasite vaccine antigens. In theory, organoids derived from parasite exposed, infected, or hyper-immune individuals could be applied in a similar way to screen the immunogenicity of native parasite antigens, for example by sequencing B cell genetic rear-rangements or determining specific lymphocyte responses following antigen stimulation, using single cell-based analyses. Alternatively, organoid models and adapted organoid-immune cell co-cultures could be applied to identify parasite antigens or adjuvants with immunomodulatory activity against diverse host cell types, including mucosal/surface cells and lymphocytes.

#### 4.2.3 Organoids for innate immune profiling and TLR stimulation

Mucosal organoids have also been shown to express host surface receptors that recognise parasite molecular patterns, such as toll-like receptors (TLRs) (Smith et al., 2021). TLR stimulation leads to the induction of an innate immune response through the activation of nuclear factor kappa B (NF-kB), resulting in the release of pro-inflammatory cytokines and chemokines (Liu et al., 2017). Kayisoglu et al. (2021) have shown that when human and murine gastric and intestinal organoids are stimulated with lipopolysac-charide (LPS), a molecule found on the outer membrane of gram-negative bacteria and known ligand of TLR4, *CXCL2* (the murine functional homologue of *IL-8*) expression is induced. These results highlight the functionality of organoids as potential models for screening novel adjuvants (including parasite-derived molecules with adjuvant potential) that aim to induce favourable pro-inflammatory responses, particularly at mucosal surfaces, since stimulants can be readily applied to the apical surface of relevant organoid models and scaled to a 96-well plate format.

#### 4.2.4 Antibody neutralisation and host protection assays

In the context of vaccine development, organoids have potential as an effective platform for investigating antibody neutralization. For example, Carrera Montoya et al. (2024) used ALI-differentiated human nasal epithelium (HNE) organoids to accurately model SARS-CoV-2 infection in the upper respiratory tract. When ALI-HNE organoids were treated with sera from mice vaccinated with a SARS-CoV-2 virus like particle (VLP) vaccine, complete neutralisation of the viral infection was observed (Carrera Montoya et al., 2024). In parasitology, organoids could be used in a similar manner to identify whether antibody neutralization of a target antigen/s is sufficient to prevent parasite invasion, life stage development, or to block antigen activity on host cells.

#### 4.2.5 Modelling vaccine delivery via M−cell uptake pathways

Finally, organoids can also be used to model vaccine uptake, particularly at mucosal surfaces. Microfold cells (M−cells) are specialised epithelial cells found primarily in the follicle-associated epithelium of Peyer’s patches in the SI (Mabbott et al., 2013). They play a vital role in the host’s immune response to GI parasites by sampling antigens in the gut lumen and transporting them across the epithelium to immune cells such as dendritic cells and macro-phages in the underlying mucosal tissue. As various parasites infect the GI tract, a vaccine exploiting the natural M−cell uptake path-ways could be successful in ensuring parasitic antigens are delivered to immune-inductive sites, akin to a natural infection. Whilst M−cells are not inherently found in intestinal organoid models, various studies have shown that their presence can be induced by the addition of Receptor Activator of Nuclear Factor-kB (RANK-L) (de Lau et al., 2012; Rouch et al., 2016; Yin and Zhou, 2018). Tong et al. (2023) set out to test a vaccine delivery system suitable for orally administered vaccines that would exploit M cell uptake pathways. They successfully reported the uptake of nanocarrier delivery vehicles across 2D monolayered murine intestinal organoids, facilitated by r1 protein and mediated by the M cells transcytosis pathway (Diller et al., 2020; Tong et al., 2020). This provided proof-of-concept of the utility of organoids for modelling vaccine uptake at mucosal surfaces that are common sites of parasite infection. Overall, the various approaches outlined here for modelling different components of vaccine development, from antigen discovery to adjuvant performance and delivery system uptake, demonstrate the promising potential of organoids as a tool in streamlining anti-parasite vaccine development.

### 4.3 Integrating advanced technologies into 3D parasitology models

The full potential of multicellular 2D and 3D cell culture models in parasitology will only be realised through the integration of complementary technologies that enhance biological relevance, scalability, and functional readout. In particular, immune cell co-culture systems and genetic manipulation technologies, such as CRISPR-Cas9, offer transformative tools to dissect host-parasite interactions, profile immune responses, and model genetically defined resistance or susceptibility traits. While these approaches have been explored extensively in human disease contexts, their application in parasitology remains nascent. Below, we outline two key areas poised for future development.

#### 4.3.1 Organoid-immune and stromal cell co-culture

Integrating organoids with other advanced systems, such as immune or stromal cell co-cultures, organ-on-a-chip platforms, and bioengineered scaffolds, will further expand the utility of these models and better mimic the *in vivo* microenvironment. Co-culture with immune cells enables more comprehensive investigations of how parasites and their products modulate host responses, including cytokine release, barrier disruption, and immune evasion strategies. Stromal cells, including fibroblasts, mesenchymal stem cells, and pericytes, provide structural and biochemical support to epithelial tissues and play critical roles in tissue repair, inflammation, and fibrosis −processes that are frequently subverted or dysregulated during parasitic infection. Incorporating stromal components into organoid systems can recreate important epithelial-stromal crosstalk and allow exploration of how parasites or their secreted products manipulate the local tissue milieu to establish infection or induce pathology. This is particularly relevant for helminth infections that elicit strong tissue remodelling responses or protozoan parasites that persist in fibrotic or hypoxic niches. Together, immune and stromal cell co-cultures can improve the fidelity of 3D models, offering deeper insight into host-pathogen dynamics while reducing reliance on animal experimentation, consistent with the 3Rs. Further details of organoid-ILC co-culture are described in Perez et al. (2025a) and López-Sandoval et al. (2025).

#### 4.3.2 Genetic manipulation of 3D culture systems

To determine the functional importance of specific genes within organoids, such as those involved in organoid differentiation or host-parasite interactions, gene silencing or knockout technologies can be used. Several approaches have been adapted to introduce DNA or RNA into organoids (Menche and Farin, 2021; Teriyapirom et al., 2021). The simplest method employs the use of short interfering (si) RNA to transiently knockdown organoid genes of interest using the cell’s RNAi gene silencing machinery to degrade endogenous mRNAs. siRNAs can be introduced into 2D or 3D organoids by incubation and mRNA knockdown can occur within hours, which is reported to be considerably enhanced by the presence of 10 % serum (Morgan et al., 2018). For stable gene knockdown, use of short hairpin (sh) RNA is effective. Lentiviral vectors expressing shRNA can be transduced into organoids, with inclusion of an antibiotic resistance gene enabling selection and subsequent expansion. Moreover, shRNA expression can be controlled using tetracycline-inducible vectors.

As an alternative to RNAi, CRISPR-Cas9 gene editing can be applied to organoids, adapting technology developed in other research fields. CRISPR-Cas9 can be used for gene knockdown or over-expression, as well as for generation of reporter organoids expressing transgenes, such as GFP or tdTomato, to identify and monitor specific cell types and follow specific cell lineages (Menche and Farin, 2021; Sun et al., 2021). The application of CRISPR-Cas9-mediated genome editing in organoid systems was first demonstrated in 2013, when Schwank et al. (2013) successfully employed homology-directed repair (HDR) to correct the *CFTR* locus in murine intestinal organoids derived from cystic fibrosis patients, resulting in enhanced cAMP-induced swelling phenotypes. Since this study, CRISPR-Cas9 has been extensively adopted and adapted across the organoid field, with numerous comprehensive reviews detailing its advancements and methodological refinements (Artegiani et al., 2020; Hendriks et al., 2020; Geurts et al., 2023; Hendriks et al., 2023).

Despite this progress, the targeted use of CRISPR-Cas9 for investigating host-pathogen interactions within organoid models remains largely under-utilised. This technology presents a particularly useful tool to “unlock” research involving non-traditional host model species in which the generation of genetically modified hosts from which primary cells could be derived is not feasible. Instead, the application of CRISPR-Cas9 provides an accessible approach to genetically modify organoid and spheroid lines to suit specific parasite systems, enabling mechanistic investigation models for the species of interest.

Antibody-mediated detection of cell marker genes is widely used to define organoid cell types and developmental states. However, the use of transgenic reporter mice or CRISPR-Cas9 knock-in facilitates the generation of organoids with fluorescently tagged marker proteins, enabling detection in real time and without the need for antibodies. This approach was used to detect the expansion of tuft cells in murine SI organoids, in which the dTomato reporter was expressed under the control of the tuft cell-associated *Dclk* promoter (Perez et al., 2025b). Transgenic knock-in would be particularly useful in cell culture systems of non-model host species, for which commercial antibodies used to detect specific host antigens can have limited cross-reactivity.

The introduction of genetic elements into organoid cultures has been previously achieved via both viral (such as adenovirus or lentivirus) and non-viral (electroporation or lipofectamine) based approaches. When testing the efficacy of viral vectors for transfection of iPSC-derived lung organoids, Belova et al. (2025) found that the adenoviral vectors rAdV5 and rAAV9 were capable of generating stable transductions of both basal and secretory cells with more than 90 % efficacy. Many studies have utilised electroporation transfections, with Skoufou-Papoutsaki et al. (2023) demonstrating upwards of 98 % transfection efficacy following the introduction of a synthetic gRNA alongside Cas9 enzyme for targeted gene knockouts of duodenal, ileal, and colon organoids. Transfection efficacy can vary widely, however, depending on the organoid tissue type. Artegiani et al. (2020) compared HDR and non-homologous end joining (NHEJ) based techniques for integration of *mNEON* into ductal and hepatocyte organoids. Although clonal *mNEON*-expressing lines were established, neither method consistently achieved robust genetic modification. NHEJ was more efficient at integration of genetic material when compared to HDR, however this yielded only a 5 % success rate (Artegiani et al., 2020). NHEJ- and HDR-based methods were comparable in terms of efficacy when tested in intestinal organoids, however neither method yielded more than 30 % successful integration (Artegiani et al., 2020).

CRISPR-Cas9-based genome-wide screening offers an additional powerful modality, enabling systematic interrogation of gene function at scale. Unlike conventional CRISPR editing, that targets individual loci via a single specific guide RNA (gRNA), CRISPR screens deploy wider gRNA libraries across pooled cell populations, allowing for high-throughput assessment of gene essentiality and phenotypic outcomes (Bock et al., 2022). Such approaches have been successfully applied to intestinal, hepatic, cerebral, and renal organoid models, elucidating critical pathways involved in cell differentiation, survival, and organogenesis (Ungricht et al., 2022; Li et al., 2023; Liang et al., 2023). For example, Hansen et al. (2023) employed CRISPR screening in foetal SI organoids to identify *Smar-ca4* and *Smarcc1* as key regulators maintaining a progenitor-like state. Beyond developmental processes, CRISPR-based screening has also been applied to the study of viral pathogenesis. Large-scale genomic screens in organoids have identified host factors essential for infection by a range of viruses, including SARS-CoV-2, herpes simplex virus type 1 (HSV-1), parainfluenza virus, and measles virus (Porotto et al., 2019; Beumer et al., 2021; Bellizzi et al., 2024). Although this approach has not been used in the context of a parasite infection, the potential utility of this screening platform could be extended to the field of parasitology, where it could be used to unravel host pathways involved in parasite development, invasion and immune modulation. With emerging data continuing to highlight the ability of organoids to facilitate the *in vitro* development of parasites that are historically challenging to maintain in culture, the ability to genetically modify the host system represents a promising future avenue to dissect host-parasite interactions in a contextually relevant setting.

While protozoan parasites such as *T. gondii* have been extensively targeted using CRISPR-Cas9 to elucidate their mechanisms of host invasion and persistence (Shen et al., 2014; Shen et al., 2017; Young et al., 2019), efforts to apply similar strategies to multicellular helminths remain limited. The *in vitro* culture of helminths is inherently challenging, and CRISPR-based genetic manipulation of these organisms has had minimal success (McVeigh, 2020; Lalawmpuii and Lalrinkima, 2023). In this context, engineering the host environment via organoid modification offers a compelling alternative, enabling researchers to explore mechanisms of host-parasite interacting molecules from the host side.

## 5 Conclusion and future directions

Multicellular 2D and 3D culture systems are already transforming parasitology research, offering powerful and ethically aligned tools to investigate parasite development, host interactions, and treatment response *in vitro*. These models provide scalable, host-specific platforms that bridge the gap between traditional 2D cell culture and *in vivo* experimentation. While technical challenges remain, the coming decade is likely to see continued expansion in the application of these models driven by improvements in culture media standardisation, tissue engineering technology, and access to well-characterised biobanks. Co-culture systems incorporating immune or stromal cells, as well as advanced imaging and omics technologies, will offer increasingly nuanced insights into host-parasite dynamics.

Genetic tools such as CRISPR-Cas9 will further enable targeted modification of host cells to dissect host-parasite interacting molecules to robustly underpin specific mechanisms of infection. High-throughput drug and vaccine screening platforms will benefit from these advances, facilitating early-stage testing of candidate therapeutics and contributing to the discovery of novel intervention targets. For veterinary parasitology, the development and sharing of livestock-derived organoid models remain a key priority to enable comparative and translational research. As these tools continue to mature, they will offer the field of parasitology new opportunities to reduce reliance on animal models, improve model predictiveness, and accelerate innovation in parasite control and treatment.

## Figures and Tables

**Table 1:** Applications of multicellular 2D and 3D culture systems in protozoan research. References provided in order of host species then tissue source and are listed within these categories by date (earliest to most recent).

Culture system	Protozoan parasite	Research focus	Study design/key findings	Reference
Human (3D brain microvessel model)	*Plasmodium falciparum (egress products derived from parasite-infected red blood cells)*	Effect of parasite-infected cell products on brain microvasculature	Endothelial structural loss, reduced microvilli, loss of endothelial junction markers	Korbmacher et al., 2025
Human (cerebral organoids)	*Toxoplasma gondii*	Tachyzoite to bradyzoite stage differentiation	Induction of innate immune responses, cell type-specific invasion and putative bradyzoite differentiation	Seo et al., 2020
Human (cerebral organoids)	*Trypanosoma brucei gambiense*	Establish an *in vitro* model to study the molecular basis of sleeping sickness	Transcriptomic analysis revealed a mixed cell-mediated pro-inflammatory (type 1, type 2 and type 17) immune response.	Chandrasegaran et al., 2023
Human (cortical brain organoids)	*P. falciparum (parasite not directly modelled)*	Model of heme-mediated brain injury associated with cerebral malaria	Heme treatment induced increases in the chemokine CXCL10 and its receptor CXCR3 and apoptosis, attenuated by NRG-1.	Harbuzariu et al., 2019
Human (cortical brain organoids)	*T. gondii*	Delivery of host protein exogenously expressed by *T. gondii*	Deposition of GRA16-fused host protein expressed in T. gondii, into infected neuronal cells and associated transcriptomic shift in host organoid cells.	Bracha et al., 2024
Human (cholangiocyte HepG2/C3A spheroids)	*P. falciparum*	*In vitro* cultivation of liver stage parasites	Development of sporozoites into schizonts and subsequently merozoites, which were infective to red blood cells, linking liver to blood stage parasite cultivation *in vitro*.	Caygill et al., 2025
Human (small intestinal organoids)	*Cryptosporidium parvum*	Cultivation through parasite life cycle *in vitro*	Microinjection of *C. parvum* sporozoites into the lumen of small intestinal organoids facilitated parasite stage differentiation and production of infective oocysts.	Heo et al., 2018
Human (intestinal ALI cultures)	*C. parvum*	Cultivation through parasite life cycle *in vitro*	Parasite life cycle cultivation in an open-format model and the ability to generate genetic crossover strains using this model.	Wilke et al., 2019
Human (retinal organoids)	*T. gondii*	Establish an *in vitro* model of ocular toxoplasmosis	Live and heat-killed parasites induce inflammatory responses, including IL-6, CXCL8 and CXCL10, but this is more highly sustained by live parasites.	Ashander et al., 2025
Murine (hepatic organoid and monocyte co-culture)	*Leishmania infantum*	Determine differences in host responses to parasite challenge depending on 3D culture system format	Up-regulation of proinflammatory cytokines only when challenged organoids were co-cultured with monocytes. Responses increased in suspension culture compared to embedded culture.	Lütkemeyer, 2024
Bovine (3D brain and heart microvessels)	*Trypanosoma congolense*	Sequestration of *T. congolense *parasites to the vessel wall	Successful sequestration and microfilament formation. Variations in sequestration rates for different strains in different tissue-type microvessel cultures.	Porqueddu et al., 2025
Feline (small intestinal 2D liquid interface cultures)	*T. gondii*	Cultivation of parasite sexual stages *in vitro*	Parasite sexual stages limited to the feline gut *in vivo* could be established *in vitro* and that linoleic acid is key to this process	Martorelli Di Genova et al., 2019
Chicken (small intestinal organoids)	*Eimeria tenella*	Cultivation through parasite life cycle *in vitro*	Development of schizonts, gametes and oocysts *in vitro*	Teng et al., 2025

**Table 2:** Applications of multicellular 2D and 3D culture systems in helminth research. References provided in order of host species then tissue source and are listed within these categories by date (earliest to most recent).

Culture system	Helminth parasite	Research focus	Study design/key findings	Reference
Human (cholangiocytes, H69)	*Clonorchis sinensis*	Effect of ES products	Spheroids in SpheroFilm microwells co-cultured with ES for 5 and 10 days. Modulation of immune and extracellular matrix genes.	Kim et al., 2021
Human (colon)	*Anisakis* spp.	Effect of *Anisakis* EVs on epithelial cells	2D organoids treated with L3 EVsevery 24 h (two administrations). Modulation of genes involved in cell cycle, apoptosis and immune response.	Bellini et al., 2024
Human (hepatoma HepG2 cells)	*Fasciola hepatica*	*In vitro* parasite-host interaction model	Physical interaction of newly excysted juveniles with spheroids results in the development of tegumental spines and sensory structures.	Vitkauskaite et al., 2025
Murine (SI)	*Nippostrongylus brasiliensis*	Uptake of *N. brasiliensis* EVs	Microinjection into lumen of 3D organoids. EV internalisation is an active process (reduced at 4 °C)	Eichenberger et al., 2018
Murine (colon)	*Trichuris muris*	Uptake of *T. muris* EVs	Microinjection into lumen of 3D organoids. EV internalisation is an active process (reduced at 4 °C)	Eichenberger et al., 2018
Murine (caecum)	*T. muris*	Effect of *T. muris* EVs on epithelial cells	Microinjection into lumen of 3D organoids. EV-mediated down-regulation of viral response-associated genes.	Duque-Correa et al., 2020
Murine (caecum)	*T. muris*	L1 invasion of organoids	2D organoids (caecaloids) co-cultured with L1 larvae. L1 degradation of mucus layer and syncytial tunnel formation	Duque-Correa et al., 2022
Murine (caecum)	*T. muris*	Epithelial cell invasion by *T. muris* L1 larvae	Bioengineered 2D organoids (caecaloids) for bilateral access using microfluidics. Live imaging of syncytial tunnel formation and cellular responses to invasion.	Hofer et al., 2024
Murine (SI)	*Heligmosomoides polygyrus*	Effect of larvae in stem cells	Granuloma-associated crypt cells generated fetal-like “spheroids” in culture. Intestinal stem cell markers were lost, despite continued epithelial proliferation. Granuloma-associated Lgr5 – crypt epithelia activated an IFN- γ dependent transcriptional program, highlighted by Sca-1	Nusse et al., 2018
Murine (SI)	*Trichinella spiralis*	Effect of ES on tuft cells	3D tuft cell reporter organoids (Trpm5-LacZ). Transient increase in intracellualr Ca2 + following stimulation with Ts extract or ES.	Luo et al., 2019
Murine (SI)	*H. polygyrus*	Effect of ES and L3 on organoid response to IL-13 and IL-4	3D organoids treated with IL-4 and IL-13. Suppression of cytokine-induced secretory cell differentiation in presence of ES or L3; ES-induced spheroid formation.	Drurey et al., 2021
Murine (SI)	*H. polygyrus bakeri*	Effect of infection or ES products on organoid stem cells	3D organoids post-infection or treated with IL-4 and IL-13. Infection or ES-induced reprogramming of intestinal stem cells	Karo-Atar et al., 2022
Murine (SI)	*Heligmosomoides polygyrus bakeri*	Basal and apical cell response to L4 and adult parasites, ES and EVs	2D organoids (enteroids). Differential effects on apical and basal cell gene expression; cell specific internalisation of EVs and associated up-regulation of interferon-stimulated genes	White et al., 2024
Murine (SI)	*Haemonchus contortus*	Effect of ES products and microRNA mimic on epithelial cell differentiation	3D tuft cell reporter organoids (Dclk-tdTomato) treated with IL-13. Adult ES or miRNA mimic-mediated suppression of secretory cell differentiation.	Perez et al., 2025
Murine (Liver)	*Echinococcus multilocularis* larvae	Hepatocyte-based metacestode culture	3D culture of hepatocytes and mesenchymal stem cells using collagen scaffold. De-differentiation of protoscolesces into infective vesicles within 8 weeks.	Li et al., 2018
Ovine (gastric and Ileal)	*Teladorsagia circumcincta*	Invasion of organoids by *T. circumcincta* L3	3D organoids. Burrowing of L3 larvaethrough matrigel and epithelial cells, and survival in organoid lumen.	Smith et al., 2021
Ovine (gastric)	*H. contortus*	Effect of ES and microRNA mimic on epithelial cell differentiation	3D organoids treated with IL-13. Adult ES or miRNA mimic-mediated suppression of secretory cell differentiation.	Perez et al., 2025b
Bovine (gastric)	*Ostertagia ostergagi*	Effect of *O. ostergagi* L3 and ES products	3D organoids. Rapid organoid ’ballooning’ on exposure to L3 or ES, possible osmotic effect.	Faber et al., 2022
Equine (jejunum)	*Strongylus vulgaris*, cyathostomins and *Parascaris univalens*	Effect of parasite L3 on epithelial cell morphology and gene expression	2D organoids (enteroids) with basolateral IL-4 and IL-13 exposure. No invasion of organoid cells by apically-applied L3 but changes in cell morphology.	Hellman et al., 2024
Porcine (SI)	*Trichinella spiralis* (L1)	Co-existence mechanisms of porcine epidemic diarrea virus (PEDV) and *T. spiralis*	3D organoids co-cultured with PEDV and *T. spirali*s L1 or ES. Suppression of PEDV-induced inflammatory cytokines and reduced tissue damage in presence of L1 or ES.	Liu et al., 2024
Canine (SI)	*Ascaris suum*	Uptake of *A. suum* adult female EVs	3D organoids co-cultured with EVs. EV uptake and presence in lumen within 24 h.	Chandra et al., 2019

## Glossary

### Abbreviations

2D Caecaloids: Multicellular monolayers structures derived from caecal epithelial stem cells, used to study host-parasite interactions specific to the caecum, such as those with *T. muris*.
Adult Stem Cells (ASCs): Tissue-resident stem cells capable of self-renewal and generating organ-specific differentiated cell types. They are commonly used to derive organoids that mimic adult tissues *in vitro*.
Air-Liquid Interface (ALI): A culture technique where tissue fragments or organoid cells are exposed to air on one side and nutrients on the other, promoting epithelial differentiation and better mimicking *in vivo* environments like the lung or intestine.
Assembloids: Advanced multicellular systems formed by combining different organoids or incorporating additional cell types (e.g., immune, stromal, neuronal), providing a more complete model of organ systems or inter-tissue interactions.
Bioprinting Techniques: 3D printing-based methods to spatially organize cells, ECM components, or spheroids into defined architectures. Used to engineer more structured or organ-like 3D culture systems.
Cystic Structure: A spherical organoid-like formation lacking typical budding or crypt structures, often resulting from exposure to parasite products. Associated with epithelial dedifferentiation or altered stem cell states.
Differentiation Media: Culture medium formulated to promote maturation and lineage commitment of organoid cells, supporting the development of cell types found in differentiated tissues (e.g., enterocytes, goblet cells).
Early Infection Gap: A poorly studied phase of infection immediately after parasite entry but before mature stages develop. This stage is critical for pathogenesis and is often inaccessible *in vivo*, but can now be modelled *in vitro* using organoids or spheroids.
Embedded Cultures: Organoids or spheroids cultured within a semi-solid ECM-like matrix (e.g., Matrigel), enabling 3D growth and polarity but potentially limiting access to the apical surface or diffusion of substances.
Enteroid: A type of organoid derived specifically from intestinal epithelium, often referring to small intestinal models (duodenum, jejunum, ileum). Used widely to study gut biology and parasitic infections.
Embryonic Stem Cells (ESCs): Pluripotent stem cells derived from early embryos, capable of differentiating into all three germ layers. Occasionally used to generate organoids mimicking developmental stages or for disease modelling.
Expansive Growth Media: Culture medium enriched with growth factors to promote proliferation and maintenance of stemness in organoid cultures, often used to expand organoid lines before differentiation.
Extracellular Matrix (ECM): A complex network of proteins and polysaccharides (e.g., laminin, collagen) that provides structural support and biochemical signals to cells in 3D cultures. Often mimicked using materials like Matrigel.
Hanging Drop Cultures: A scaffold-free spheroid generation technique where cells are aggregated by gravity in droplets suspended on inverted plate lids, allowing for uniform and reproducible spheroid formation.
Hydrogels: Water-rich polymer-based matrices used as ECM mimetics to support 3D cell culture. They can be natural (e.g., collagen, alginate) or synthetic, with tunable stiffness and porosity.
Low-Attachment Plates: Culture plates treated to prevent cell adhesion, allowing suspended cells to self-aggregate into spheroids without attachment to the surface, facilitating scaffold-free 3D culture.
Magnetic Levitation: A scaffold-free technique where cells are loaded with magnetic nanoparticles and levitated into 3D aggregates using magnetic fields, enabling controlled spheroid assembly.
Multicellular 2D Monolayers: Monolayers composed of multiple epithelial cell types derived from dissociated organoids. These are seeded on flat surfaces or transwells and maintain cellular heterogeneity and polarity.
Organoids: Self-organising 3D multicellular structures derived from stem cells (ASCs, ESCs, or iPSCs) that recapitulate key features of tissue architecture and function. Widely used in parasitology to study infection, immune responses, and drug effects.
Organismoids: Highly engineered 3D culture platforms that combine multiple cell types, organoids, microfluidics, or vascular components to mimic whole-organ or systemic functions, with enhanced physiological relevance.
Organs-on-a-Chip: microfluidic devices incorporating living cells (often organoid-derived) into miniature tissue-like structures with dynamic flow, enabling real-time observation of interactions and drug responses under near-physiological conditions.
Pluripotent Stem Cells (iPSCs): Genetically reprogrammed cells capable of differentiating into any cell type. Induced pluripotent stem cells are used to create organoids with patient-specific or developmental relevance.
Rotating Wall Vessel Bioreactors: A scaffold-free 3D culture method in which cells are maintained in constant suspension via gentle rotation, promoting uniform spheroid formation and mimicking low-shear conditions found *in vivo*.
Spheroids: 3D cellular aggregates formed from one or more cell types without a supporting scaffold. They mimic some aspects of tissue architecture and cell– cell interactions, often used for toxicity assays, pathogens infection models, or drug screening.
Submerged Culture: A common organoid culture technique where organoid-containing ECM domes are fully immersed in growth medium. Suitable for many tissue types but with limitations in oxygenation or access to the apical surface.
Suspension Cultures: Cultures where organoids or spheroids are grown freely in liquid medium without matrix embedding. This format facilitates apical exposure and is often used in infection studies or immune co-cultures.
Three Rs (3Rs): Ethical principles in animal research: *Replacement* (use of alternatives to animals), *Reduction* (minimising animal use), and *Refinement* (improving animal welfare). 3D models such as organoids support the 3Rs by reducing reliance on animal models.
Tissue Engineering (TE) Models: Bioengineered *in vitro* systems that integrate cells, scaffolds, and bioactive factors to recreate functional tissues. TE models are used to study parasitic infections in more realistic, organ-like settings.
